# Lacrimal syringing

**Published:** 2009-06

**Authors:** Sue Stevens

**Affiliations:** Former Nurse Advisor, *Community Eye Health Journal*, International Centre for Eye Health, London School of Hygiene and Tropical Medicine, Keppel Street, London WC1E 7HT, UK.

**Figure:**
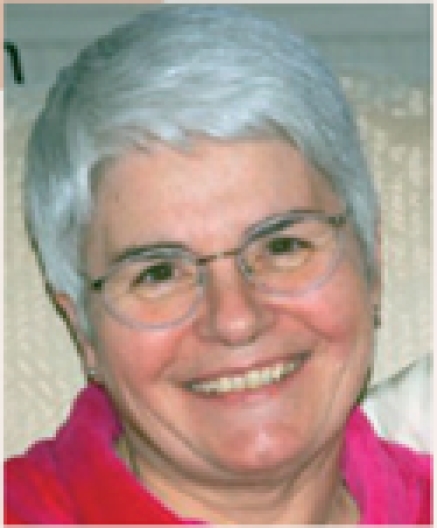


## Before performing any eye procedure

- Wash your hands (and afterwards too).
- Position the patient comfortably with head supported.
- Minimise distractions, both for yourself and the patient.
- Ensure good lighting.
- Always explain to the patient (and any companion, if appropriate) what you are going to do.

## Reasons for lacrimal syringing

- to check the naso-lacrimal passage for any blockage
- to flush out debris, e.g., lacrimal passage infection.

This technique is also suitable for administering antibiotics to the lacrimal passages and for introducing dye for X-ray procedures.

## You will need

- a torch (held by an assistant) or preferably a well-powered lamp
- magnification (e.g., loupes)
- normal saline
- a sterile 2 ml syringe
- a sterile Nettleship dilator
- a sterile lacrimal cannula
- local anaesthetic eye drops
- clean cotton wool or gauze swabs
- a towel
- gloves

**Note:** The pictures do not show gloves being worn; however, the wearing of gloves for all clinical procedures is now mandatory in most centres.

## Preparation

It is important that the anatomy of the lacrimal apparatus is understood before carrying out this procedure (Figure 1).

**Figure:**
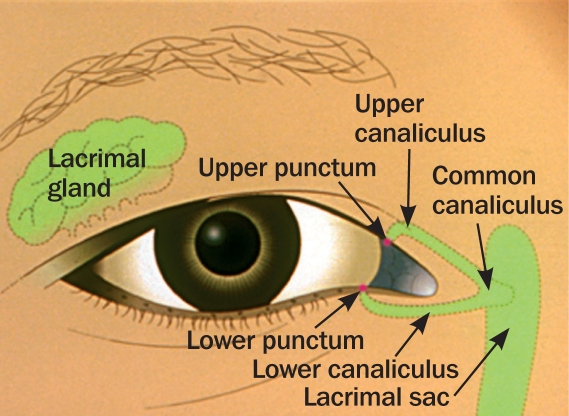
Figure 1.

- Position the adult patient lying down with head supported on a pillow, or sitting with head against the high back of a chair.
- If the patient is a child, you may need to ask your assistant to wrap the child in a sheet and gently restrain the child throughout the procedure.
- Place the towel across the patient's neck to absorb any fluid spillage.
- Check the Nettleship dilator and do not use if there is any damage to the tip.
- With the syringe, draw up about 1 ml of saline and then attach it to the cannula (Figure 2).
- Flush the cannula with a small amount of saline to ensure it is patent.

**Figure:**
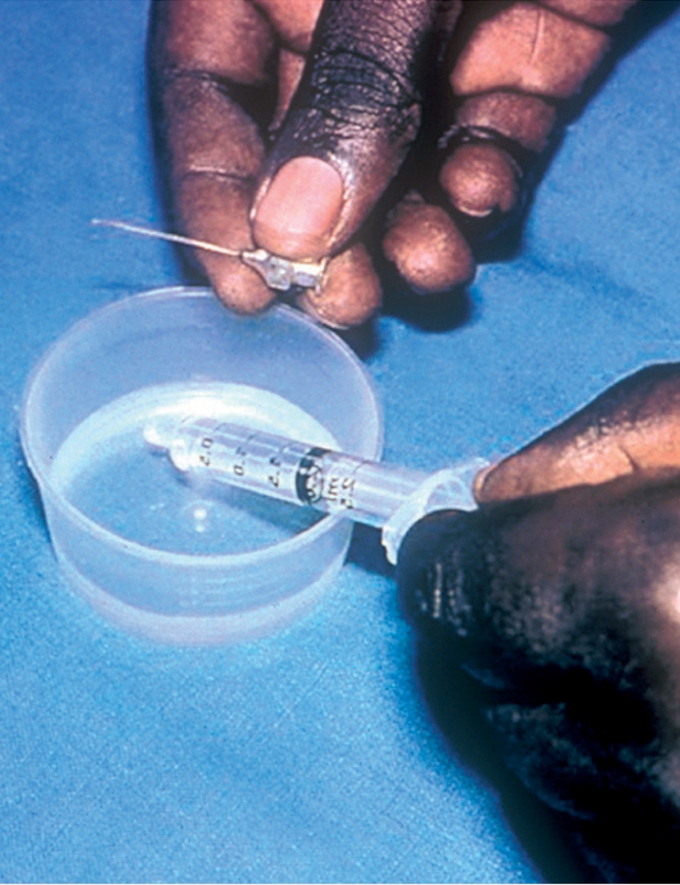
Figure 2.

## Method

- Instil the local anaesthetic eye drops, allowing the drops to fall directly over the lower punctum, and wait about 30 seconds.
- Ask the patient to look upwards and outwards (away from the nose) and to maintain this gaze until the procedure is over.
- With cotton wool or a gauze swab, gently pull down the lower eyelid to expose the lower punctum.
- With the other hand, insert the Nettleship dilator into the lower punctum, following the direction of the lower canaliculus (Figure 3). Gently rotate it a few times and then withdraw the dilator (this dilation will facilitate the insertion of the cannula).
- Take the syringe containing the saline and attached cannula and insert the cannula tip into the lower punctum (Figure 4).
- Inject the fluid slowly, and explain to the patient that they may have the sensation of a salty taste at the back of the mouth and the need to swallow fluid.
- If the patient is not aware of this sensation, it indicates a blockage somewhere in the lacrimal apparatus. The fluid may be seen coming through the upper punctum.

**Figure:**
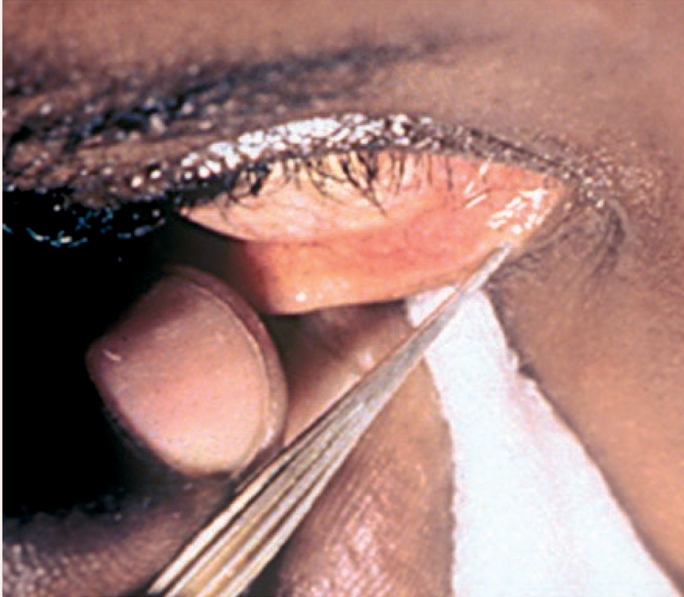
Figure 3.

**Figure:**
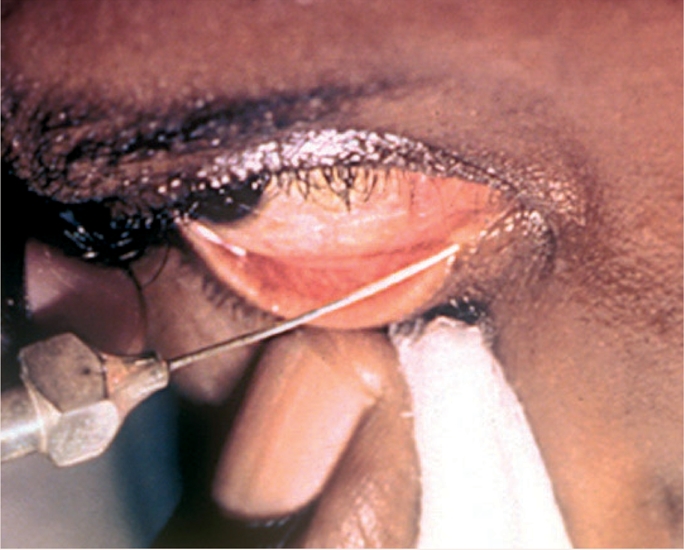
Figure 4.

The next part of the procedure requires two people:

- Instil the local anaesthetic drops directly over the upper punctum and again over the lower punctum; wait about thirty seconds.
- Gently raise the upper eyelid to expose the upper punctum.
- Occlude the upper punctum with the Nettleship dilator. The assistant will need to hold the dilator in the upper punctum while the syringing is repeated through the lower punctum as before (Figure 5).
- If the patient still does not experience the salty taste and swallow sensation, this will indicate that the site of the blockage is in the common canaliculus or the lacrimal sac.

**Figure:**
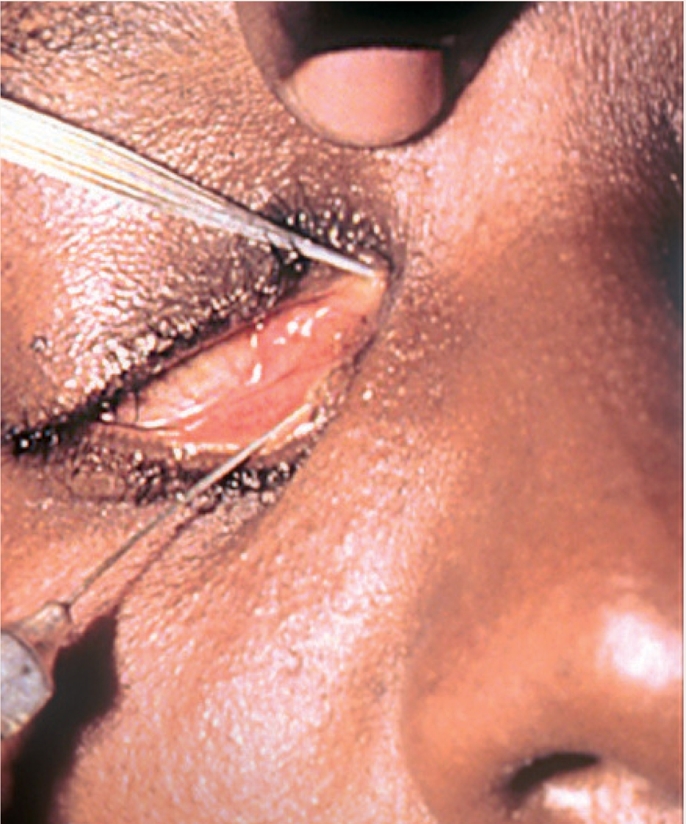
Figure 5.

Record the findings in the patient's documentation.

